# Construction of a Risk Prediction Model of Swallowing Dysfunction During Recovery from Acute Ischemic Stroke

**DOI:** 10.3390/healthcare14142100

**Published:** 2026-07-14

**Authors:** Xinyu Zhang, Huaqiang Li, Xiangwen Hao, Yanyang Li, Bianying Feng, Jingqi Chen, Zhen Dan, Ailipinai Yasen, Xiaoyan Zheng, Anren Zhang, Qiuhong Man

**Affiliations:** 1 School of Health Preservation and Rehabilitation, Chengdu University of Traditional Chinese Medicine, Chengdu 610072, China; 2024ks625@stu.cdutcm.edu.cn (X.Z.); lihuaqiang@stu.cdutcm.edu.cn (H.L.); 2 Department of Clinical Laboratory, Shanghai Fourth People’s Hospital, School of Medicine, Tongji University, Shanghai 200060, China; haoxiangwen@tongji.edu.cn (X.H.); 2510916@tongji.edu.cn (Y.L.); 2180283@tongji.edu.cn (B.F.); jingqichen@tongji.edu.cn (J.C.); 2605000@tongji.edu.cn (X.Z.); 3 Rehabilitation Medicine Center, The First Affiliated Hospital of Nanjing Medical University, Nanjing 210029, China; emmaching2001@gmail.com (Z.D.); 18645753884@163.com (A.Y.); 4 Department of Rehabilitation Medicine, Shanghai Fourth People’s Hospital Affiliated to Tongji University, Shanghai 200060, China

**Keywords:** acute ischemic stroke, swallowing dysfunction, predictive modeling, biomarker

## Abstract

**Objective:** This study aimed to analyze risk factors for post-stroke dysphagia during the recovery phase of acute ischemic stroke and to construct a preliminary risk prediction model for patients who were eligible for bedside swallowing assessment. **Methods:** A retrospective analysis was performed for patients with ischemic stroke who were continuously admitted to Shanghai Fourth People’s Hospital. Swallowing function was assessed using the water swallowing test. Univariate and multivariate analyses were used to screen variables, and a predictive model was constructed using binary logistic regression. The model was visualized using a nomogram. Calibration curves and receiver operating characteristic curves were employed to assess the model’s accuracy and predictive value, while decision curve analysis was used to examine the model’s clinical relevance. **Results:** Overall, 209 ischemic stroke patients were included in the analysis. Univariate and multivariate logistic regression analyses identified higher NIHSS score and lower triglyceride level as independent factors associated with swallowing dysfunction (*p*-value < 0.05); the full model (NIHSS + TG) yielded AUCs of 0.685 (95% CI: 0.523–0.783) in the training set and 0.679 (95% CI: 0.606–0.700) in the test set, with corresponding sensitivities of 0.52 and 0.61, and specificities of 0.78 and 0.70, respectively. Compared with NIHSS alone (AUC 0.652, 95% CI: 0.518–0.746), the AUC improvement was not significant (DeLong *p* = 0.09), but the net reclassification improvement was 0.26 (95% CI: 0.02–0.49). **Conclusions:** This preliminary derivation model, based on NIHSS and triglyceride levels, demonstrates modest discrimination but provides incremental reclassification value over NIHSS alone for estimating the risk of PSD in cooperative patients. The model should be considered exploratory, and external validation is required before any clinical application.

## 1. Introduction

Although the incidence of ischemic stroke (IS) has shown a decreasing trend in recent years, the risk of stroke over a lifetime has increased due to population aging. From 1990 to 2019, the global number of IS-related deaths increased from 2.04 million to 3.29 million, and it is projected to rise further to 4.9 million by 2030 [1,2]. Post-stroke dysphagia (PSD) is a common complication following stroke, with symptoms such as excessive salivation, coughing while drinking, aspiration, and choking during meals. The incidence of PSD is as high as 78% [3]. Although swallowing function may improve during the recovery phase after a stroke, up to half of the cases progress to a chronic condition, leading to a range of complications such as psychological disorders, aspiration pneumonia, and choking. These severely impact patients’ quality of life [4] and may result in poor prognosis or even death [5]. Research has shown that early screening for swallowing function in stroke patients, coupled with early intervention, can help reduce hospital stay duration, lower hospitalization costs, and decrease mortality. This approach is one of the key methods for reducing complications and improving outcomes [6,7,8,9]. Therefore, early screening, identification, and intervention for PSD are of great significance [10].

Currently, commonly used diagnostic and screening tools for post-stroke dysphagia (PSD), such as swallowing radiography and the Water Swallowing Test (WST), require patients to have a relatively good physiological condition in order to be performed [11]. More importantly, the swallowing assessment scales commonly used in clinical practice are highly subjective, and differences in evaluation criteria among healthcare providers can affect the accuracy of the swallowing results [12]. Therefore, for patients with poor compliance to clinical swallowing function tests, combining subjective scales with objective diagnostic results can help improve the accuracy of clinical decision-making. Although recent studies have focused on rehabilitation interventions, swallowing muscle physiology, and instrumental assessment techniques for post-stroke dysphagia, few studies have developed simple and clinically applicable risk prediction models using routinely available laboratory biomarkers during the recovery phase of ischemic stroke [4,10,13].

Therefore, objective clinical and laboratory indicators may provide complementary information for early risk stratification before or alongside bedside swallowing assessment. However, because the present study included only patients who were able to undergo the Water Swallowing Test and excluded patients with impaired consciousness or tracheostomy, the model was developed for cooperative patients with acute ischemic stroke and should not be extrapolated to patients who are comatose, mechanically ventilated, tracheostomized, or unable to complete swallowing assessment. The present study aimed to establish a preliminary risk prediction model integrating clinical severity and routinely available laboratory biomarkers to support early identification of patients at increased risk of post-stroke dysphagia.

## 2. Material and Methods

### 2.1. Subjects

The study population includes patients who were consecutively admitted to the Department of Cerebrovascular Disease and the Department of Rehabilitation Medicine at Shanghai Fourth People’s Hospital, affiliated with Tongji University, from December 2021 to October 2024, with acute ischemic stroke. Swallowing function was assessed within 24 h after admission using the 30 mL Water Swallowing Test (WST) [14], which has been used as a bedside screening tool for swallowing impairment and aspiration risk in patients with stroke [15]. According to the five-grade WST profile, grade 1 indicates drinking all water in one gulp without choking within the expected time and was defined as normal swallowing; grades 2–5 indicate suspected or definite swallowing abnormality, such as repeated gulps, choking, or inability to complete drinking, and were used to define PSD for risk-stratification purposes [14,16,17]. The WST was performed by trained rehabilitation physicians or nurses using a standardized bedside procedure. Because the outcome was defined using the WST, the derivation cohort was restricted to patients who were able to complete this bedside assessment. A retrospective analysis of all patients’ medical records was conducted.

Inclusion criteria were as follows: (1) age ≥ 18 years; (2) diagnosis of acute ischemic stroke based on clinical manifestations and brain MRI findings; (3) completion of swallowing function assessment using the 30 mL WST within 24 h after admission; (4) ability to cooperate with bedside swallowing assessment; Exclusion criteria were as follows: (1) presence of neurological diseases that could cause swallowing dysfunction; (2) tracheostomy; (3) impaired consciousness; (4) pre-stroke dysphagia; (5) lack of brain MRI records; and (6) follow-up duration of less than 14 days among patients with poor swallowing recovery during model construction.

Therefore, the derivation cohort consisted of patients with acute ischemic stroke who were able to cooperate with bedside swallowing assessment. Among the participants, 119 patients were classified into the swallowing dysfunction group, and 90 patients were in the normal swallowing function group. The study was approved by the ethics committee of Shanghai Fourth People’s Hospital (Ethics Approval No. 2024236-001) (2025-01-13 00:00:00) and registered with the China Clinical Trial Center (Registration No. CTR2500095810). Since this was a retrospective study, exemption from informed consent was applied.

### 2.2. Imaging Assessment

All patients underwent brain MRI with DWI sequences at admission using a 3.0T MRI scanner (Siemens Magnetom Prisma, Siemens Healthineers, Erlangen, Germany). Two experienced neuroradiologists, blinded to clinical and swallowing data, independently reviewed the images and recorded the following lesion characteristics: (1) brainstem involvement (yes/no), defined as visible DWI hyperintensity in the midbrain, pons, or medulla oblongata; (2) bilateral hemispheric involvement (yes/no), defined as DWI lesions in both cerebral hemispheres; and (3) lesion multiplicity, classified as single versus multiple (≥2 distinct DWI hyperintense lesions). Disagreements were resolved by consensus.

### 2.3. Predictors and Outcomes

Clinical and laboratory information for participants was collected from the electronic medical record system. The data included the following details: (1) demographic information (gender and age); (2) clinical admission data (underlying diseases and NIHSS scores); (3) laboratory biomarkers within 48 h of admission (routine complete blood count, liver and kidney function parameters, myocardial markers, coagulation function parameters, inflammatory indices, and inflammatory cytokines). Data were entered into Excel (version 2019, Microsoft Corp., Redmond, WA, USA) and double-checked by two researchers to establish the database. The resulting data were then imported into SPSS 26.0 (IBM Corp., Armonk, NY, USA) for statistical analysis.

### 2.4. Statistical Methodology

Electronic medical records were reviewed, and data were entered into Excel and double-checked by two researchers to establish the database. For missing data, a threshold of 5% was applied. Variables with missing data exceeding 5% were excluded, while variables with missing data ≤ 5% were handled using multiple imputation with the R package “mice” (version 3.16.0) within R version 4.3.2 (R Foundation for Statistical Computing, Vienna, Austria) to generate reliable imputed values for analysis. Specifically, albumin (missing 11.2%), prealbumin (missing 18.7%), body mass index (missing 9.8%), and lipid-lowering therapy data (missing 15.3%) were excluded because their missing proportions exceeded the pre-specified threshold of 5%. Variables with missing data ≤ 5%, including triglycerides (missing 2.9%) and CRP (missing 1.4%). The resulting data were then imported into SPSS 26.0 for statistical analysis.

Regarding sample size, the present study followed the Events Per Variable (EPV) guideline recommended for logistic regression-based prediction models. With 119 PSD events and 8 candidate predictors in the overall cohort, the EPV was approximately 14.9, exceeding the minimum recommended threshold of 10 events per variable [18,19]. Therefore, the sample size was considered adequate for the exploratory model development in this derivation cohort.

For continuous variables, normality tests were performed. Data that followed a normal distribution were expressed as mean ± standard deviation (x ± s), and group comparisons were conducted using an independent samples *t*-test. For non-normally distributed data, the median (interquartile range) M (P25, P75) was used, and group comparisons were made using the Wilcoxon rank-sum test. Categorical data were expressed as frequency (n, %) and compared using the chi-square (χ^2^) test. Variables that showed statistical significance in univariate analysis were included in a multivariate binary logistic regression model to identify key predictors. A Nomogram was constructed, and the model’s performance was evaluated using receiver operating characteristic (ROC) curves, calibration curves, and Decision Curve Analysis (DCA) through R 4.3.2.

The proposed model has low computational complexity because it is based on a binary logistic regression equation with only two predictors: NIHSS score and triglyceride level. For each patient, the model only requires multiplication of the regression coefficients by the corresponding predictor values, summation of these terms, and conversion of the linear predictor into a predicted probability using the logistic function. Therefore, the prediction process is computationally simple and can be implemented using a bedside calculator, spreadsheet, or electronic medical record system without specialized hardware. Model development was also computationally lightweight because of the small sample size and limited number of predictors.

## 3. Results

### 3.1. Demographics and Clinical Characteristics

**Figure 1:**
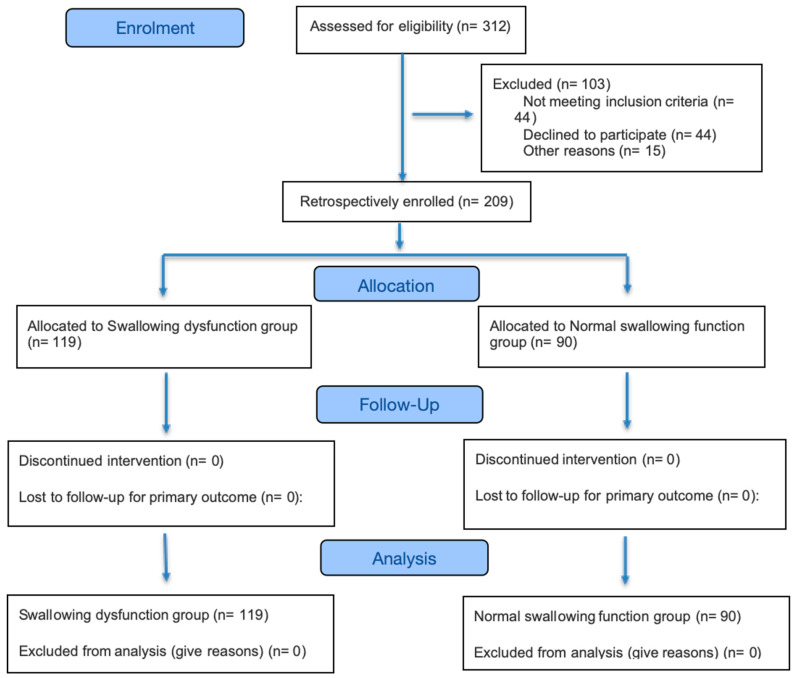
Flow diagram.

A total of 119 patients (56.9%) were in the swallowing dysfunction group, and 90 patients (43.06%) were in the non-swallowing dysfunction group. See Figure 1. In the swallowing dysfunction group, there were 79 males and 40 females, with a history of hypertension in 73 cases, diabetes in 52 cases, and stroke in 24 cases. In the non-swallowing dysfunction group, there were 55 males and 35 females, with a history of hypertension in 51 cases, diabetes in 27 cases, and stroke in 7 cases. A comparison of general data between the two groups revealed that the swallowing dysfunction group had significantly higher proportions of patients with a history of stroke, diabetes, and surgery, as well as older age and higher NIHSS scores at admission compared to the non-swallowing dysfunction group (*p*-value < 0.05). See Table 1 and Table 2.

### 3.2. Predictor Screening

The total population was divided into training and validation sets in a 7:3 ratio, and balance tests were conducted. The training set was used for factor selection and model construction, while the validation set was used for model validation. The occurrence of swallowing dysfunction in patients with acute ischemic stroke during hospitalization was set as the dependent variable. Eight variables with statistical significance in the univariate analysis of baseline data and laboratory results were selected for inclusion in the multivariate binary logistic regression analysis. These variables included: age, NIHSS score at admission, history of cerebral infarction, C-reactive protein, triglycerides, international normalized ratio (INR), lymphocyte count, and neutrophil count. Multivariable logistic regression showed that higher NIHSS score (OR = 1.120, 95% CI: 1.000–1.255; *p*-value < 0.05) and lower triglyceride level (OR = 0.521, 95% CI: 0.286–0.948; *p*-value < 0.05) were independently associated with swallowing dysfunction. The results are shown in Table 3, Table 4 and Table 5.

### 3.3. Construction of the Risk Prediction Nomogram for Swallowing Dysfunction After Acute Ischemic Stroke

**Figure 2:**
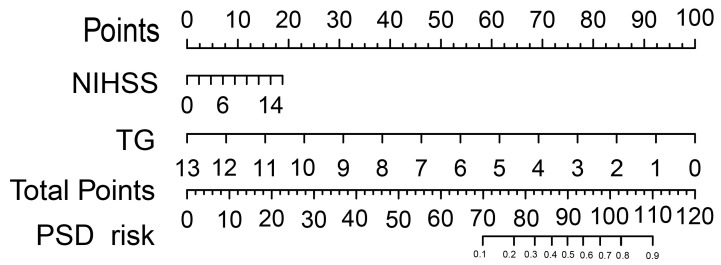
Nomogram.

The logistic regression analysis indicated that NIHSS score and triglyceride level were independently associated with swallowing dysfunction after ischemic stroke. Specifically, higher NIHSS score and lower triglyceride level were associated with increased PSD risk. Subsequently, R 4.3.2 software was used to create a nomogram for visualizing the predictive model, as shown in Figure 2. The effect of each variable on the occurrence of swallowing dysfunction after stroke is represented by a score. A vertical line is drawn from the value on the axis to intersect with the score axis, where the point of intersection corresponds to the score for that variable. The total score is obtained by summing the scores for each variable, and the predicted probability of swallowing dysfunction in stroke patients is determined by the total score.

### 3.4. Risk Prediction Model Validation

The predictive ability of the model was evaluated using ROC curve analysis. The model demonstrated modest predictive ability (training AUC: 0.685, sensitivity: 0.52, specificity: 0.78; internal test AUC: 0.679, sensitivity: 0.61, specificity: 0.70). Compared with an NIHSS-only reference model (AUC 0.652), the full model showed no significant AUC improvement (DeLong *p*-value = 0.09), though NRI was 0.26 (95% CI: 0.02–0.49) and IDI was 0.03 (95% CI: −0.01 to 0.07) (Table 6). Adding imaging characteristics (brainstem involvement, bilateral lesions, multiplicity) did not improve performance (DeLong *p*-value = 0.45; Table 7). Since none of the imaging variables reached statistical significance in multivariate analysis, the final parsimonious model was retained with NIHSS and triglycerides only.

**Figure 3:**
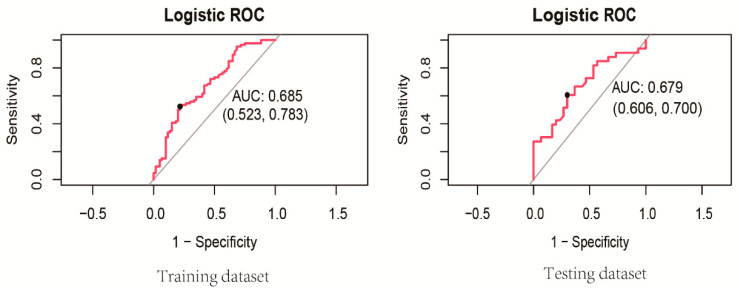
ROC curve.

**Figure 4:**
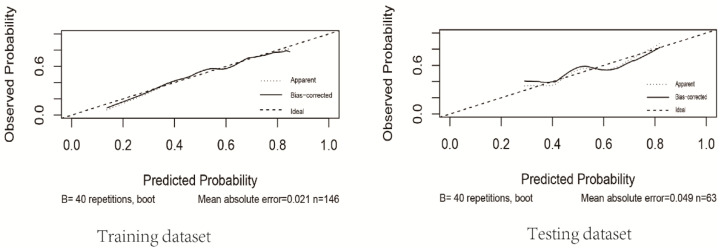
Calibration curve.

**Figure 5:**
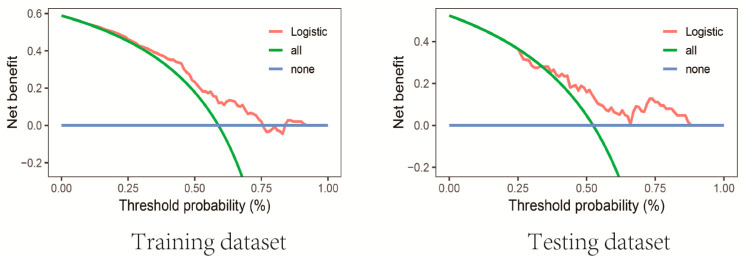
Clinical decision curve.

Decision Curve Analysis (DCA) was conducted to assess whether patients could benefit from the model in clinical practice. When the threshold probability ranged from 0.1 to 1, the decision-making based on the predictive model was beneficial to patients. The calibration curve indicated that the model’s actual predictions closely aligned with the ideal predictions. Quantitatively, the Brier score was 0.212 in the training set and 0.224 in the validation set, indicating acceptable overall prediction error. The mean absolute errors between predicted and observed probabilities were 0.021 and 0.049, respectively, further supporting good calibration. See Figure 3, Figure 4 and Figure 5.

## 4. Discussion

The primary cause of swallowing dysfunction in stroke patients is cerebrovascular events, such as cerebral hemorrhage or ischemic stroke. These events result in damage to the nervous system that controls swallowing function [20,21], which subsequently affects the transmission of neural signals to the muscles of the mouth and throat, leading to muscle coordination dysfunction and impaired swallowing [22,23]. Studies have found that the more severe the neurological injury, the higher the incidence of swallowing dysfunction [24]. The NIHSS is used in clinical practice to assess the severity of stroke, with higher scores indicating more severe neurological deficits [25]. Although the NIHSS does not include specific items related to swallowing function, clinical studies have shown that NIHSS scores can predict PSD and be used for early swallowing dysfunction assessment, with moderate sensitivity and specificity [26]. Rebecca D et al. [27] also found that an NIHSS score greater than 9 and a FIM score less than 55 can be used for early screening of swallowing dysfunction. Although the sensitivity and specificity of NIHSS for predicting swallowing dysfunction are lower than those of FIM, it is more widely applied in clinical settings. Therefore, the NIHSS score, in addition to being a measure of stroke severity, can also be considered a reliable predictor of the presence of swallowing dysfunction [28]. Our study results are consistent with previous findings, with the median NIHSS score for the swallowing dysfunction group in acute ischemic stroke patients being 4, and the median NIHSS score for the non-swallowing dysfunction group being 2.5. The mean NIHSS score of the swallowing dysfunction group was significantly higher than that of the non-swallowing dysfunction group (*p*-value < 0.001). After adjusting for confounding factors in multivariate logistic regression analysis, we found that the NIHSS score was an independent risk factor for swallowing dysfunction after acute ischemic stroke.

Triglycerides are an important stored energy source and are closely related to glucose metabolism, blood pressure, and liver function. In the present study, lower triglyceride levels were associated with a higher risk of PSD. This inverse association should not be interpreted as a causal protective effect of triglycerides. Rather, low triglyceride levels may reflect premorbid nutritional depletion, reduced energy intake, acute metabolic stress, or systemic catabolism after stroke [29,30,31,32]. Acute ischemic stroke may induce stress-related hormonal changes, increased protein breakdown, negative nitrogen balance, impaired gastrointestinal function, and elevated resting metabolic rate, all of which may contribute to nutritional depletion and poorer functional recovery [27,28,33,34,35]. Malnutrition and insufficient nutritional reserve may impair swallowing-related muscle function and recovery capacity, thereby increasing the risk of dysphagia [36]. Therefore, triglycerides in this model should be considered a potential surrogate marker of nutritional or metabolic status rather than a direct mechanistic factor.

Our comparative analysis revealed that while the addition of triglyceride levels did not significantly improve the AUC compared with NIHSS alone at the population level (DeLong *p*-value = 0.09), the Net Reclassification Improvement (NRI) of 0.26 (95% CI: 0.02–0.49) suggests that TG may offer modest incremental value in individual-level risk stratification. This apparent discrepancy between population-level AUC and individual-level NRI is not uncommon in prediction modeling: a biomarker that does not substantially shift the overall ROC curve may still help reclassify patients who fall near the decision threshold, particularly those with intermediate NIHSS scores. The NRI of 0.26 indicates that approximately one-quarter of patients were correctly reassigned to higher- or lower-risk categories when TG was incorporated. Importantly, the NIHSS alone—even with the widely cited cutoff of ≥9 proposed by Jeyaseelan et al. [27]—has limited predictive performance for PSD, with reported sensitivities ranging from 68.3% to 75% and specificities from 61.5% to 62%. In our cohort, the NIHSS-only model achieved an AUC of 0.652, consistent with recently published data showing an NIHSS AUC of 0.663 for PSD prediction. The modest improvement offered by TG, while not dramatic, represents a step toward incorporating readily available laboratory biomarkers into PSD risk stratification—a direction that aligns with the growing recognition that malnutrition and metabolic status, as reflected by low TG levels, are important determinants of post-stroke outcomes.

Because albumin, prealbumin, body mass index, GNRI, CONUT score, and lipid-lowering therapy were not consistently available in this retrospective dataset, residual confounding and reverse causality cannot be excluded [37]. Future studies should incorporate comprehensive nutritional assessments to clarify whether triglycerides provide independent predictive information beyond established nutritional indices.

In addition to these clinical and laboratory factors, the role of neuroanatomical lesion characteristics deserves further consideration. Although lesion topography is widely recognized as a key determinant of post-stroke dysphagia [38,39], our multivariate analysis did not identify brainstem involvement, bilateral lesions, or lesion multiplicity as independent predictors in this cohort. This may be explained by several factors. First, the overall frequency of brainstem lesions was low (4.8%), limiting statistical power. Second, NIHSS score, which remained significant in the adjusted model, may serve as a composite proxy for overall infarct burden, partially capturing the effect of lesion characteristics [27,40]. Third, our cohort included only patients who could undergo MRI and cooperate with the water swallowing test, which may have excluded the most severely affected individuals with critical brainstem or bilateral lesions. Future studies with larger sample sizes and quantitative lesion mapping (e.g., voxel-based lesion-symptom mapping) are warranted to further clarify the neuroanatomical predictors of PSD [41,42].

Currently, the commonly used Vachon drinking test and other assessment scales evaluate swallowing function by observing the patient’s swallowing actions and performance during drinking [43]. However, these tests have certain subjectivity and limitations, as factors such as the patient’s subjective experience and the evaluator’s level of expertise can influence the results. In contrast, the PSD prediction model based on blood and clinical characteristics uses objective and quantifiable blood markers to predict swallowing function, minimizing the impact of subjective factors [44,45]. Therefore, combining the Vachon drinking test with the prediction model can complement each other, improving the accuracy and applicability of the assessment across different patient populations. The combination of both methods not only allows for a more accurate determination of the patient’s swallowing function status but also provides a more reliable basis for subsequent rehabilitation treatment [10]. By integrating these two assessment methods, clinicians can gain a more comprehensive understanding of the patient’s swallowing function and potential risk factors, helping to develop a more personalized treatment plan, which may improve treatment outcomes and the patient’s quality of life [13,46]. It should be emphasized that the present model was derived from patients who were able to complete the Water Swallowing Test. Therefore, its intended use is early risk stratification in cooperative patients with acute ischemic stroke, rather than diagnosis of dysphagia in patients who cannot undergo bedside swallowing assessment. Patients with impaired consciousness, tracheostomy, mechanical ventilation, or severe cognitive impairment may have different clinical characteristics, including sedation exposure, enteral feeding, systemic inflammation, respiratory compromise, and different NIHSS distributions. These factors may alter both neurological severity scores and metabolic biomarkers. Accordingly, the present model should not be extrapolated to these excluded populations without separate validation.

## 5. Conclusions

We developed a preliminary predictive model based on NIHSS score and triglyceride levels to estimate the risk of swallowing dysfunction during the recovery phase of acute ischemic stroke. The model showed modest discrimination and may assist early PSD risk stratification in cooperative patients eligible for bedside swallowing assessment. The model should be considered exploratory, and external validation is required before any clinical application.

## 6. Limitations

1. This study is a single-center retrospective analysis and did not use an external cohort for validation. 2. This study employed the Water Swallowing Test (WST) as the diagnostic criterion for swallowing dysfunction, rather than swallowing fluoroscopy, which may have affected the accuracy of the swallowing assessment to some extent. 3. Only basic imaging features were assessed, without detailed lesion segmentation, volumetric analysis, or evaluation of key swallowing-related cortical regions. 4. Patients unable to complete bedside swallowing assessment were excluded; therefore, the model should not be applied to patients with impaired consciousness, severe cognitive impairment, tracheostomy, or mechanical ventilation without separate validation. 5. Because triglyceride levels may reflect nutritional or metabolic status and comprehensive nutritional indicators were unavailable, residual confounding and reverse causality cannot be excluded.

## Figures and Tables

**Table 1:** Baseline characteristics of the included total population.

Characteristic	Total (n = 209)	PSD (n = 119)	PSND (n = 90)	*p*-Value
Age	71.14 ± 9.89	72.66 ± 9.94	69.13 ± 9.51	<0.05 *
NIHSS	4 (2–6.5)	5 (2–8)	3 (1–5)	<0.05 *
Male	134	79	55	>0.05
Female	75	40	35	>0.05
Coronary artery disease	13	8	5	>0.05
Hypertension	124	73	51	>0.05
Diabetes	79	52	27	<0.05 *
History of stroke	31	24	7	<0.05 *
Arrhythmia	11	8	3	>0.05
Brainstem involvement, n (%)	10 (4.8)	7 (5.9)	3 (3.3)	0.392
Bilateral lesions, n (%)	23 (11.0)	15 (12.6)	8 (8.9)	0.393
Multiple lesions, n (%)	82 (39.2)	52 (43.7)	30 (33.3)	0.129

*p*-value < 0.05 was considered statistically significant. Values are presented as mean ± standard deviation, median (interquartile range), or frequency (percentage) as appropriate. *p*-values were derived from independent samples *t*-test, Wilcoxon rank-sum test, or chi-square (χ^2^) test, as appropriate. Abbreviations: PSD, post-stroke dysphagia; PSND, post-stroke non-dysphagia; NIHSS, National Institutes of Health Stroke Scale, *, *p* < 0.05. Note: *p*-values are rounded to two decimal places.

**Table 2:** Baseline laboratory results for the included population.

Characteristic	Total (n = 209)	PSD (n = 119)	PSND (n = 90)	*p*-Value
HbA1c	6.6 (5.8–8.45)	6.8 (5.8–8.8)	6.35 (5.8–8.025)	>0.05
FIB	3.9 (3.38–4.38)	3.95 (3.5–4.43)	3.82 (3.27–4.23)	>0.05
TG	1.4 (1.07, 1.77)	1.26 (1.06–1.68)	1.45 (1.09–1.88)	>0.05
LDL	3.17 ± 1.03	3.08 ± 0.96	3.30 ± 1.12	>0.05
CRP	2.72 (1.06–6.61)	3.18 (1.27–8.55)	2.29 (0.85–4.33)	>0.05
HB	144 (134–152.5)	144 (134–152)	144 (133–153.5)	>0.05
INR	1.02 (0.97–1.07)	1.03 (0.98–1.1)	1.01 (0.96–1.04)	<0.05 *
APTT	29.8 (27.9–32.8)	30 (28–33)	29 (27.88–32.65)	>0.05
Mono	0.38 (0.29–0.49)	0.36 (0.29–0.48)	0.39 (0.29–0.51)	>0.05
cTnI	0.01 (0.01–0.02)	0.01 (0.01–0.02)	0.01 (0.01–0.02)	>0.05
sCr	70 (58.85–90.35)	71.1 (58.7–91.5)	69.4 (58.93–84.05)	>0.05
Mb	45.82 (28.34–84.18)	50.19 (31.66–90.34)	37.64 (26–61.18)	<0.05 *
CKI	2 (1.26–3.43)	2.1 (1.34–3.51)	1.89 (1.19–3.40)	>0.05
ALP	79.86 (65.5–96.71)	83.61 (70.3–99.26)	76.21 (61.04–91.48)	<0.05 *
LYM	1.38 (1.03–1.95)	1.29 (0.99–1.71)	1.53 (1.09–2.11)	<0.05 *
UREA	5.63 (4.79–7.19)	5.82 (4.75–7.59)	5.44 (4.89–7.01)	>0.05
MCHC	30.8 (29.6–31.65)	30.7 (29.6–32.8)	30.85 (29.68–31.9)	>0.05
Glu	8.1 (6.4–12.15)	8.8 (6.7–12.6)	7.25 (6.3–11.5)	>0.05
Baso	0.02 (0.01–0.03)	0.02 (0.01–0.03)	0.02 (0.01–0.03)	>0.05
NEUT	5.12 (3.93–6.59)	5.24 (3.98–6.93)	4.72 (3.84–5.76)	<0.05 *
TBil	13.07 (9.56–17.75)	13.36 (10.81–18.97)	12.22 (8.34–16.9)	<0.05 *
TP	75.96 (71.98–80.20)	76.05 (71.98–81.24)	75.91 (71.93–79.82)	>0.05

HbA1c: glycated hemoglobin; FIB: Fibrinogen; TP: total protein; TG: Triglyceride LDL: Low-Density Lipoprotein; CRP: C-reactive protein; HB: Hemoglobin; sCr: serum creatinine; INR: international normalized ratio; APTT: activated partial thromboplastin time; NEUT: neutrophil; Mono: Monocyte counts; cTnI: cardiac troponin; Mb: Myoglobin; CKI: creatine kinase isoenzymes; ALP: Alkaline phosphatase; UREA: carbamide; MCHC: mean corpuscular hemoglobin concentration; Glu: glucose; Baso: Basophil count; LYM: lymphocytes; TBil: total bilirubin, *, *p* < 0.05. Note: *p*-values are rounded to two decimal places.

**Table 3:** Analysis of Demographic and Clinical Characterization Outcomes of the Training Set.

Characteristic	PSD (n = 86)	PSND (n = 60)	*p*-Value
Age	72 (66–78.25)	68 (64–75)	<0.05 *
NIHSS	4 (2–8)	2.5 (1–4.75)	<0.05 *
Sex (Male)	62	38	>0.05
Coronary artery Disease	7	4	>0.05
Hypertension	54	36	>0.05
Diabetes	39	21	>0.05
History of stroke	16	4	<0.05 *
Arrhythmia	5	2	>0.05

*, *p* < 0.05. Note: *p*-values are rounded to two decimal places.

**Table 4:** Analysis of training set lab results.

Characteristic	PSD (n = 86)	PSND (n = 60)	*p*-Value
HbA1c	6.9 (5.9–8.8)	6.4 (5.73–7.95)	>0.05
FIB	3.87 (3.48–4.37)	3.85 (3.33–4.31)	>0.05
TG	1.23 (1.06–1.67)	1.51 (1.2–2.01)	<0.05 *
CRP	3.19 (1.21–8.86)	2.49 (0.88–4.48)	<0.05 *
HB	144.5 (134–151.25)	145.5 (135–155.25)	>0.05
INR	1.035 (0.97–1.09)	1.00 (0.96–1.45)	<0.05 *
APTT	30.10 (28.08–32.5)	29.95 (28–33.4)	>0.05
Mono	0.36 (0.28–0.49)	0.38 (0.31–0.49)	>0.05
cTnI	0.01 (0.01–0.02)	0.01 (0.01–0.03)	>0.05
sCr	71.2 (57.65–92.6)	69.50 (60.7–83.35)	>0.05
Mb	47.32 (28.37–90.88)	37.64 (26.65–59.58)	>0.05
CKI	1.965 (1.29–3.28)	2.13 (1.29–3.90)	>0.05
ALP	80.35 (67.12–97.34)	75.17 (61.09–89.86)	>0.05
LYM	1.29 (0.98–1.74)	1.53 (1.10–2.04)	<0.05 *
UREA	5.83 (4.65–8.24)	5.50 (5.12–7.08)	>0.05
MCHC	30.8 (29.6–31.5)	30.3 (29.63–31.75)	>0.05
Glu	8.6 (6.78–12.38)	7.2 (6.33–11.35)	>0.05
Baso	0.02 (0.01–0.02)	0.02 (0.01–0.03)	>0.05
NEUT	5.41 (4.00–6.99)	4.72 (3.89–5.65)	<0.05 *
TBil	13.07 (10.79–18.35)	12.32 (8.55–16.51)	>0.05
TP	75.96 ± 7.70	76.04 ± 5.94	>0.05
LDL	3.05 ± 0.95	3.32 ± 1.14	>0.05

HbA1c: glycated hemoglobin; FIB: Fibrinogen; TP: total protein; TG: Triglyceride; CRP: C-reactive protein; LDL: Low-Density Lipoprotein; HB: Hemoglobin; sCr: serum creatinine; INR: international normalized ratio; APTT: activated partial thromboplastin time; NEUT: neutrophil; Mono: Monocyte counts; cTnI: cardiac troponin; Mb: Myoglobin; CKI: creatine kinase isoenzymes; ALP: Alkaline phosphatase; UREA: carbamide; MCHC: mean corpuscular hemoglobin concentration; Glu: glucose; Baso: Basophil count; LYM: lymphocytes; TBil: total bilirubin, *, *p* < 0.05. Note: *p*-values are rounded to two decimal places.

**Table 5:** Binary Logistic Regression Analysis.

Characteristic	OR	95%CI	*p*-Value
Age	1.020	0.977–1.064	>0.05
NIHSS	1.120	1.00–1.255	<0.05 *
History of stroke	1.786	0.520–6.135	>0.05
Brainstem involvement	1.15	0.45–2.95	0.771
Bilateral lesions	1.08	0.58–2.01	0.807
Multiple lesions	1.22	0.72–2.07	0.459
CRP	1.042	0.987–1.101	>0.05
INR	0.615	0.018–20.913	>0.05
TG	0.521	0.286–0.948	<0.05 *
LYM	0.824	0.552–1.228	>0.05
NEUT	1.057	0.918–1.217	>0.05

CRP: C-reactive protein; TG: Triglyceride; INR: international normalized ratio; LYM: lymphocytes; NEUT: neutrophil, *, *p* < 0.05. Note: *p*-values are rounded to two decimal places.

**Table 6:** Performance comparison between the NIHSS-only reference model and the full model (NIHSS + TG).

Model	Predictors	Training AUC (95% CI)	Sensitivity	Specificity	DeLong *p*-Value	NRI (95% CI)	IDI (95% CI)
Reference model	NIHSS	0.652 (0.518–0.746)	0.48	0.80	Reference	Reference	Reference
Full model	NIHSS + TG	0.685 (0.523–0.783)	0.52	0.78	0.09	0.26 (0.02–0.49)	0.03 (−0.01–0.07)

Abbreviations: AUC, area under the curve; CI, confidence interval; NIHSS, National Institutes of Health Stroke Scale; NRI, Net Reclassification Improvement; IDI, Integrated Discrimination Improvement; TG, triglycerides.

**Table 7:** Performance of the original and imaging-adjusted models.

Model	Predictors	Training AUC	Validation AUC	Sensitivity	Specificity	DeLong *p*-Value
Original model	NIHSS + TG	0.685	0.679	0.52	0.78	Reference
Imaging-adjusted model	NIHSS + TG + brainstem + bilateral + multiple	0.687	0.682	0.52	0.78	0.45

AUC, area under the curve; NIHSS, National Institutes of Health Stroke Scale; TG, triglycerides. The original model served as the reference for the DeLong test. The imaging-adjusted model added brainstem involvement, bilateral lesions, and lesion multiplicity. No significant difference was observed between the two models (DeLong test, *p*-value = 0.45).

## Data Availability

The data presented in this study are available on request from the corresponding authors. The data are not publicly available due to privacy or ethical restrictions.
